# A case report of metastatic lung adenocarcinoma with long-term survival for over 11 years

**DOI:** 10.1097/MD.0000000000014100

**Published:** 2019-01-25

**Authors:** Tatsu Matsuzaki, Eri Iwami, Kotaro Sasahara, Aoi Kuroda, Takahiro Nakajima, Takeshi Terashima

**Affiliations:** Department of Respiratory Medicine, Tokyo Dental College, Ichikawa General Hospital, 5-11-13, Sugano, Ichikawa, Chiba, Japan.

**Keywords:** adenocarcinoma, EFGR-TKIs, *EGFR* gene mutation, long-term survival, non-small cell lung cancer, re-challenge chemotherapy, T790 M

## Abstract

**Rationale:**

This is the first known report in the English literature to describe a case of metastatic non-small cell lung cancer that has been controlled for >11 years.

**Patient concerns:**

A 71-year-old man visited our hospital because of dry cough.

**Diagnosis:**

Chest computed tomography revealed a tumor on the left lower lobe with pleural effusion, and thoracic puncture cytology indicated lung adenocarcinoma.

**Interventions:**

Four cycles of carboplatin and docetaxel chemotherapy reduced the size of the tumor; however, it increased in size after 8 months, and re-challenge chemotherapy (RC) with the same drugs was performed. Repeated RC controlled disease activity for 6 years. After the patient failed to respond to RC, erlotinib was administered for 3 years while repeating a treatment holiday to reduce side effects. The disease progressed, and epidermal growth factor receptor (*EGFR*) gene mutation analysis of cells from the pleural effusion detected the T790 M mutation. Therefore, osimertinib was administered, which has been effective for >1 year.

**Outcomes:**

The patient has survived for >11 years since the diagnosis of lung cancer.

**Lessons:**

Long-term survival may be implemented by actively repeating cytotoxic chemotherapy and EGFR-tyrosine kinase inhibitor administration.

## 1 Introduction

The prognosis of patients with advanced non-small cell lung cancer (NSCLC) is poor, and their 1-year survival rate after cytotoxic chemotherapy is only 29%.^[1]^ However, the development of epidermal growth factor receptor tyrosine kinase inhibitors (EGFR-TKIs) dramatically improves the prognosis of certain patients. Patients with EGFR-mutant advanced NSCLC receiving EGFR-TKIs have a median overall survival (OS) more than twice as long as those not receiving EGFR-TKIs (24.3 vs 10.8 months).^[2]^ The 5-year survival rate of patients with EGFR-mutant metastatic lung adenocarcinoma treated with EGFR-TKIs is 14.6%.^[3]^ However, metastatic NSCLC patients with long-term survival (>10 years) are still rare.

We treated an advanced NSCLC patient with malignant pleural effusion who survived for >11 years and for whom disease progression was controlled using drugs alone without surgery or radiation therapy.

## 2 Case presentation

**Figure 1:**
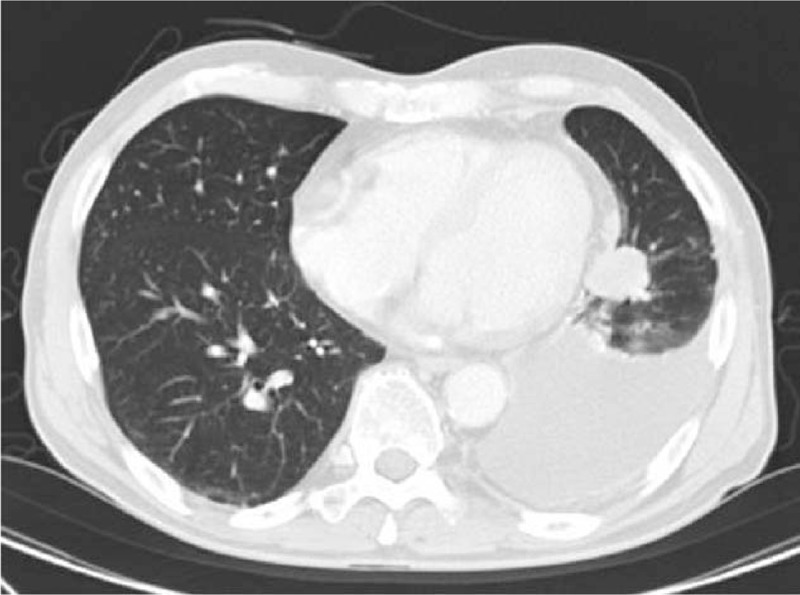
Chest computed tomography (CT) in August 2007. Chest CT scan showing a nodule in the left lower lobe with left pleural effusion.

A 71-year-old Japanese man experienced dry cough for 2 weeks and visited the Department of Respiratory Medicine at our hospital in August 2007. Enhanced chest-abdomen computed tomography revealed a tumor with a 3-cm diameter in the left lower lobe and left pleural effusion (Fig. 1). A 5-mm nodule, considered to be lung metastasis, was detected in the left upper lobe. Cytological analysis of the left pleural effusion by thoracic puncture led to the diagnosis of lung adenocarcinoma. Gadolinium-enhanced brain magnetic resonance imaging and bone scintigraphy did not reveal any other metastases. The tumor was classified as clinical T4N0M1, stage IV according to the TNM classification of the Union of International Cancer Control (UICC), 6th edition. According to the UICC 8th edition, it was classified as clinical T4N0M1a, stage IV A. The patient had a history of hypertension and was a past smoker (60 pack-years) and a company employee. The Eastern Cooperative Oncology Group performance status (ECOG-PS) at the time of admission was 1. The carcinoembryonic antigen (CEA) level was 97.4 ng/mL (normal, 0–5 ng/ml).

**Figure 2:**
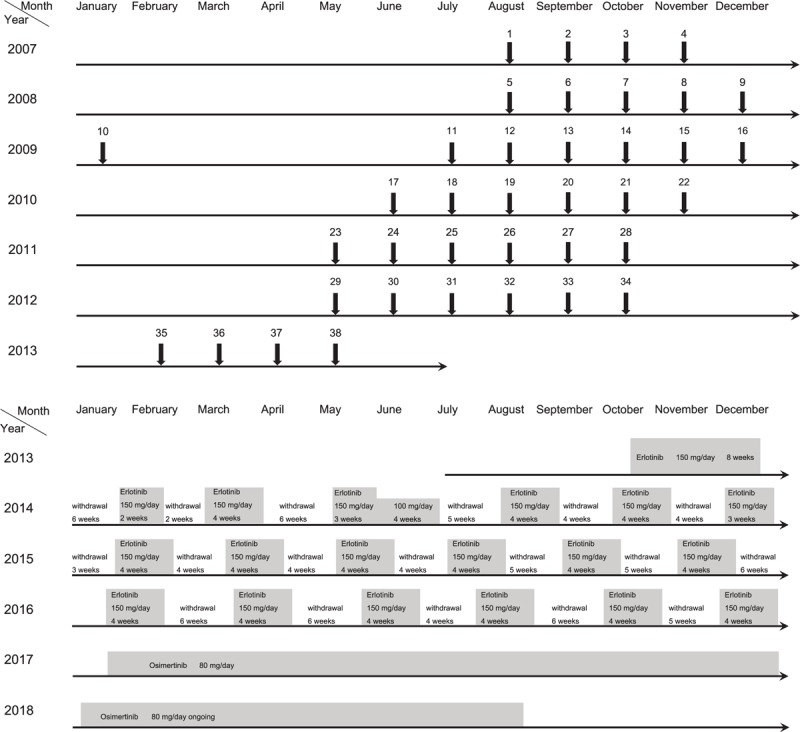
Patient's treatment course. (A) Treatment course using cytotoxic chemotherapy. Chemotherapy with a combination of carboplatin (CBDCA) and docetaxel (DTX) was performed beginning in August 2007. The tumor and pleural effusion repeatedly decreased and increased in size. After each increase, 4 to 6 cycles of the same regimen were performed until 2013, for a total of 38 cycles over 6 years. Each arrow indicates one cycle. (B) Treatment course using epidermal growth factor receptor-tyrosine kinase inhibitors (EGFR-TKIs). Administration of erlotinib began in October 2013. Skin disorders interrupted its administration. The patient interrupted and resumed treatment repeatedly. Because of the detection of the T790 M mutation in exon 20 in malignant cells from the pleural effusion, erlotinib was changed to osimertinib in January 2017, and the administration of the latter is ongoing.

Beginning in August 2007, the patient received carboplatin (CBDCA) and docetaxel (DTX). After 4 cycles, the tumor was reduced to 1 cm in diameter. The 5-mm nodule and pleural effusion had also decreased. According to the Response Evaluation Criteria in Solid Tumors version 1.1, partial response was achieved, but he experienced progressive disease (PD) after 8 months. Six cycles of re-challenge chemotherapy (RC) using the same regimen were started in August 2008 and were effective. Thereafter, at each recurrence of PD, 4 to 6 cycles of RC were administered, and by 2013, 38 cycles had been completed over 6 years of treatment (Fig. 2A). However, we could no longer control disease activity using the same chemotherapy regimen. Moreover, primary tumor size evaluation became difficult owing to massive pleural effusion; although not standard, we estimated the effect of treatment using the increase and decrease of CEA as an index. CEA increased from a minimum of 4.6 ng/ml to 33.3 ng/ml in October 2013 during repeated cytotoxic chemotherapy. Although his *EGFR* mutation status was unknown, we initiated erlotinib administration and the CEA level decreased. After 8 weeks, the patient developed grade 3 acneiform rash, assessed using the Common Terminology Criteria for Adverse Events version 5.0, and erlotinib administration was discontinued for 6 weeks. Cycles of medication and treatment holiday were repeated, and the patient was carefully observed for skin rash. Dose reduction was attempted once, but it was not effective, because we noted an elevated CEA level and intolerable skin rash. For 3 years, 4-week erlotinib administration was repeated with 4–6-week treatment holiday intervals (Fig. 2B). CEA increased from a minimum of 3.1 ng/ml to 30.4 ng/ml in January 2017 during treatment with erlotinib. We performed *EGFR* mutation analysis using adenocarcinoma cells from the pleural effusion and detected exon 19 deletion and exon 20 T790 M mutation; therefore, osimertinib was substituted for erlotinib.

We continued monthly CEA measurements after beginning osimertinib administration and noted that the level continued to decrease. In August 2018, the CEA level was 12.1 ng/ml and the ECOG-PS was 1. As of the last follow-up, the patient has survived for >11 years since the diagnosis of lung cancer.

## 3 Discussion

The clinical data of 10 patients with advanced NSCLC who survived for >5 years were retrospectively reviewed, and a good PS, adenocarcinoma, and a history of EGFR-TKI administration were the factors contributing to long-term survival.^[4]^ According to another retrospective study,^[3]^ 20 of 137 patients with EGFR-mutant lung adenocarcinoma survived for ≥5 years, and exon 19 deletion, absence of extrathoracic metastases, absence of brain metastasis, and current non-smoking status were reportedly good prognostic factors. Our case corroborated the good prognostic factors reported in these studies.

A case of metastatic NSCLC in which the patient survived for 10 years has already been reported; however, the patient underwent not only chemotherapy but also surgery and radiation therapy.^[5]^ To the best of our knowledge, ours is the first report in the English literature to describe a metastatic NSCLC case controlled for >11 years. Moreover, our patient was only treated with chemotherapy and EGFR-TKIs.

We considered that 4 treatment policies may be the key to success:

1. RC with CBDCA plus DTX;
2. repeated re-challenge erlotinib administration;
3. osimertinib administration after T790 M mutation in exon 20, which confers resistance to erlotinib; and
4. use of both cytotoxic drugs and EGFR-TKIs. We will particularly focus on the first and second policies because of the non-standard methods.

The response rate (RR) to RC of platinum doublets containing pemetrexed (PEM) or taxanes is reportedly 27.5%, with a progression-free survival (PFS) of 3.9 months and an OS of 8.7 months. This RR is high, but the PFS and OS are similar to those seen with administration of a single-agent as second-line treatment.^[6]^ Advanced NSCLC patients for whom RC with 2-drug combination therapy is performed have a longer median survival than those administered only DTX as second-line treatment.^[7]^ The current evidence that RC is superior to a single agent second-line treatment is not sufficient, but if the side effects are acceptable, RC may be a suitable option.

To safely perform RC using platinum-based 2-drug therapy, it may be necessary to include a treatment holiday period for a certain duration to facilitate physical fitness recovery. Time to progression of >3 months after ending first-line chemotherapy is a predictor of long-term survival (>2 years) in advanced NSCLC patients who receive cytotoxic chemotherapy.^[8]^ Advanced NSCLC patients who survive for >2 years have a good response to first-line cytotoxic chemotherapy, and a prolonged treatment-free interval increases long-term survival.^[9]^ In the present case, the treatment holiday after cytotoxic chemotherapy was approximately 6 months. A prolonged treatment-free interval appears to be important for restoring physical fitness; therefore, the patient could tolerate the next treatment.

A meta-analysis of randomized control studies compared cisplatin (CDDP) and CBDCA in advanced NSCLC patients. Although regimens containing CDDP did not prolong OS, subgroup analysis demonstrated that, when combined with third-generation anticancer drugs, CDDP prolonged OS more than CBDCA in patients with non-squamous cell carcinoma.^[10]^ In the TAX 326 trial, CBDCA and DTX combination therapy helped achieve an OS equivalent to that associated with CDDP and vinorelbine combination therapy; in addition, the CBDCA and DTX combination was well tolerated and facilitated a high quality of life.^[11]^ Here, 38 cycles of CBDCA and DTX combination therapy were administered. To perform platinum-based 2-drug RC, it may be advantageous to repeatedly administer CBDCA rather than CDDP to facilitate tolerability.

When erlotinib toxicity is intolerable, as in our patient who developed a severe skin rash, the dosage is generally reduced. In patients with EGFR-mutant NSCLC, dose reduction (25 mg/day) of erlotinib can reduce the toxicity while maintaining efficacy.^[12]^ In contrast, a prospective phase II trial involving low-dose erlotinib in patients with EGFR-mutant NSCLC revealed that dose reduction (50 mg/day) is not recommended because of reduced efficacy.^[13]^ Intermittent erlotinib administration on alternate days successfully maintained efficacy while reducing toxicity.^[14]^ Here, disease control was possible by RC of platinum doublets for approximately 6 cycles after the 6-month treatment holiday. Therefore, we applied the RC strategy to EGFR-TKI administration. Continued erlotinib administration was discontinued if side effects became intolerable or health-threatening and resumed when side effects disappeared. Toxicity and efficacy were balanced by allowing an approximately 4–6-week treatment holiday after the 4-week erlotinib administration. To the best of our knowledge, we were the first to employ erlotinib in RC; this method was named repeated re-challenge administration of erlotinib and was considered to alleviate suffering due to toxicity. Although there is little evidence to determine whether dose reduction, intermittent administration, or repeated re-challenge administration is better, it is important to avoid complete cessation of therapy.

The exon 20 mutation in *EGFR*, leading to the T790 M mutation, is a resistance mechanism to traditional EGFR-TKIs. Osimertinib treatment is associated with a longer median PFS than chemotherapy using PEM plus either CBDCA or CDDP as second-line treatment in T790M-mutant patients who received EGFR-TKIs.^[15]^ Our patient with the exon 19 deletion and T790 M mutation received osimertinib after erlotinib. Even if the tumor develops resistance to conventional EGFR-TKIs, it is important to recognize that drugs such as osimertinib are available and may improve long-term survival.

In EGFR-mutant advanced NSCLC with exon 19 deletion and exon 21 (L858R) mutation, patients who receive sequential therapy with erlotinib and cytotoxic drugs experience a longer median OS than those who receive cytotoxic drugs or erlotinib alone.^[16]^ It may be important to administer both EGFR-TKIs and cytotoxic drugs throughout the course of treatment.

Although more study is required, RC of CBDCA plus DTX and repeated re-challenge administration of erlotinib may be an empirical option for long-term survival. To understand the clinical and biological background of long-term survival cases, accumulation of future cases similar to ours is warranted.

## Acknowledgments

We would like to thank Editage (www.editage.jp) for English language editing.

## Author contributions

**Supervision:** Takeshi Terashima.

**Writing – original draft:** Tatsu Matsuzaki.

**Writing – review & editing:** Eri Iwami, Kotaro Sasahara, Aoi Kuroda, Takahiro Nakajima.
